# Dissociation Between Insulin Resistance and Abnormalities in Lipoprotein Particle Concentrations and Sizes in Normal-Weight Chinese Adults

**DOI:** 10.3389/fnut.2021.651199

**Published:** 2021-02-26

**Authors:** Kaare Tranæs, Cherlyn Ding, Yu Chung Chooi, Zhiling Chan, John Choo, Melvin K.-S. Leow, Faidon Magkos

**Affiliations:** ^1^Section for Obesity Research, Department of Nutrition, Exercise and Sports, University of Copenhagen, Frederiksberg, Denmark; ^2^Singapore Institute for Clinical Sciences (SICS), Agency for Science, Technology and Research (A^*^STAR) and National University Health System, Singapore, Singapore; ^3^Department of Endocrinology, Tan Tock Seng Hospital, Singapore, Singapore; ^4^Cardiovascular and Metabolic Disorders Program, Duke-NUS Medical School, Singapore, Singapore; ^5^Lee Kong Chian School of Medicine, Nanyang Technological University, Singapore, Singapore

**Keywords:** insulin resistance, lipoprotein particle size, obesity phenotypes, metabolically-unhealthy lean, nuclear magnetic resonance

## Abstract

Insulin resistance in obesity coincides with abnormalities in lipid profile and lipoprotein subclass distribution and size even before abnormalities in glucose homeostasis manifest. We aimed to assess this relationship in the absence of obesity. Insulin sensitivity (3-h intravenous glucose tolerance test and minimal modeling) and lipoprotein particle concentrations and sizes (proton nuclear magnetic resonance spectroscopy) were evaluated in 15 insulin-resistant and 15 insulin-sensitive lean Asians of Chinese descent with normal glucose tolerance, matched on age, sex, and body mass index. Despite a ~50% lower insulin sensitivity index (Si) in insulin-resistant than in insulin-sensitive subjects, which was accompanied by significantly greater acute insulin response to glucose (AIRg) and fasting insulin concentration but not different fasting glucose concentration, there were no significant differences between groups in the blood lipid profile (*p* ≥ 0.44) or the lipoprotein subclass concentrations (*p* ≥ 0.30) and particle sizes (*p* ≥ 0.43). We conclude that, contrary to observations in subjects with obesity, insulin resistance is not accompanied by unfavorable changes in the plasma lipid profile and lipoprotein particle concentrations and sizes in lean Asians with normal glucose tolerance. Therefore, insulin resistance at the level of glucose metabolism is mechanistically or temporally dissociated from lipid and lipoprotein metabolism.

**Trial Registration:** clinicaltrials.gov, NCT03264001.

## Introduction

Cardiovascular disease (CVD) remains a leading cause of death in the western world and, as the obesity epidemic spreads (1), CVD is becoming increasingly prevalent in Asia as well (2). South East Asians are particularly vulnerable to cardiometabolic disease because of greater body fat percent and more abdominal fat deposition than Caucasians at similar body mass index (BMI) (3). Central adiposity is a major risk factor for developing CVD, along with high blood pressure, increased concentrations of triglyceride and low-density lipoprotein (LDL) cholesterol, low levels of high-density lipoprotein (HDL) cholesterol, and hyperglycemia. A common link among these metabolic abnormalities, which collectively constitute the metabolic syndrome, is insulin resistance—a trait typically associated with obesity (4). However, reduced sensitivity to insulin can also be observed in the absence of obesity (5–7). Non-obese insulin-resistant individuals often present metabolic abnormalities comparable to those of obese individuals, but because they lack the telltale sign of increased body weight and adiposity, their insulin resistance remains untreated until further complications develop (7).

There is accumulating evidence suggesting that cardiometabolic disease is associated not only with traditional lipid-related risk factors (e.g., triglyceride, LDL, and HDL cholesterol concentrations), but also—and perhaps to a greater extent—with alterations in lipoprotein subclass distribution and particle size (8–10). In most studies of individuals with overweight and obesity, insulin resistance and abnormalities in lipoprotein subclass profile often coincide (11–13). In fact, alterations in lipoprotein size and particle distribution can manifest prior to the development of abnormal glucose homeostasis (10, 11, 14, 15). On the contrary, studies in non-obese individuals do not consistently find significant differences in the traditional plasma lipid profile between insulin-resistant and insulin-sensitive groups (16–18). These studies used the Homeostasis Model Assessment of Insulin Resistance (HOMA-IR) as an index of insulin sensitivity and did not evaluate lipoprotein subclass distribution and size. To gain a better understanding of the relationship between insulin resistance and plasma lipoprotein subclass profile in non-obese people, we recruited two groups of lean Asians, matched for age, sex and BMI, and evaluated insulin sensitivity by an intravenous glucose tolerance test (IVGTT) and lipoprotein subclasses by proton nuclear magnetic resonance (^1^H-NMR) spectroscopy.

## Materials and Methods

### Subjects

A total of 30 subjects (22 women and 8 men), aged 21–59 years, with a BMI between 18 and 25 kg/m^2^, participated in this study. These subjects were Singaporeans of Chinese descent and had enrolled in a larger project conducted in our laboratory (Clinical Nutrition Research Center, Singapore) between April 2017 and February 2018, which was designed to investigate the metabolic effects of negative energy balance induced by diet or exercise (19, 20). The 15 most insulin-resistant individuals on the basis of an IVGTT (section Experimental design) were matched on a one-to-one basis for sex, age, and BMI with the 15 most insulin-sensitive individuals (Table 1). Subjects were sedentary or recreationally active but untrained, and had no prior history of glucose intolerance, hypertension or dyslipidemia; all but two had normal fasting blood glucose and HbA1c concentrations at screening (one subject in each group had fasting glucose concentrations between 102 and 104 mg/dL, with normal HbA1c). Those who were using medications known to affect metabolic function (including oral contraceptives and hormone replacement therapy), using tobacco products and consuming alcohol regularly, had evidence of significant organ system dysfunction or disease, and recent weight loss or gain (≥5% change in body weight over the past 6 months) were excluded from the study. Following screening, subjects had their body composition measured by dual-energy X-ray absorptiometry and their resting metabolic rate (RMR) measured by indirect calorimetry, as previously described (21–24). Ethics approval was obtained from the Domain Specific Review Board of the National Healthcare Group in Singapore (protocol # 2016/00660), and all subjects provided their written informed consent prior to enrolment.

**Table 1:** Subject characteristics and parameters of glucose homeostasis in insulin-resistant and insulin-sensitive Asians.

	**Insulin-sensitive**	**Insulin-resistant**	***p-*value**
Age (years)	25.0 ± 5.9	27.7 ± 12.3	0.45
Sex (men/women)	4/11	4/11	–
Body mass index (kg/m^2^)	20.9 ± 2.0	21.9 ± 2.7	0.26
Body fat (%)	28.2 ± 6.6	33.3 ± 3.9	0.02
Fat mass (kg)	16.0 ± 3.7	18.9 ± 4.2	0.06
Fat-free mass (kg)	41.7 ± 9.6	37.8 ± 7.6	0.23
Resting metabolic rate (MJ/day)	5.4 ± 1.1	5.0 ± 1.0	0.33
Glucose, fasting (mmol/L)	4.94 ± 0.39	5.05 ± 0.39	0.48
Insulin, fasting (pmol/L)	37.8 ± 13.2	57.6 ± 15.6	<0.01
HOMA-IR (mU·mmol/L^2^)	1.4 ± 0.5	2.2 ± 0.6	<0.01
Insulin sensitivity, Si (L/mU·min)	3.9 ± 1.0	2.1 ± 0.5	<0.01
Insulin secretion, AIRg (mU·min/L)	816 ± 457	1,232 ± 239	<0.01
Disposition index (unitless)	2,949 ± 1414	2,577 ± 898	0.40

*Data are shown as mean ± SD for n = 15 each, and differences between groups were evaluated by Student's unpaired t-test*.

*HOMA-IR, homeostasis model assessment of insulin resistance*.

### Experimental Design

Subjects visited the laboratory in the morning (~8 a.m.) after having fasted overnight, 1 day before the actual metabolic testing day so their dietary intake and physical activity could be standardized. They were instructed to abstain from alcohol and caffeine consumption on the previous day and from performing any strenuous exercise on the preceding 3 days. During their stay (until ~8 p.m.), subjects remained in the laboratory while resting and consumed a diet designed to meet their energy requirement for weight maintenance (i.e., eucaloric diet), which was estimated for each subject by multiplying RMR (measured at screening) by a factor of 1.4 (25). Two meals (breakfast, served at 9 a.m., and dinner, served at 7 p.m.) were given to provide 60% of the total daily energy requirements, and the remaining 40% was provided by two snacks (served in the morning and afternoon). All meals and snacks were prepared in the metabolic kitchen of the Clinical Nutrition Research Center; each meal or snack contained 55% of total energy as carbohydrate, 27% as fat, and 18% as protein. Subjects were discharged after eating dinner, fasted thereafter, and returned to the laboratory the next morning (~8 a.m.) for metabolic testing. Vital signs (temperature, heart rate, and blood pressure) were obtained following 30 min of bed rest. Thereafter, fasting blood samples were collected for lipoprotein analysis, and a glucose bolus (11.4 g/m^2^ body surface area) was injected over 1, 20 min later, a single bolus of short-acting insulin (0.03 U/kg body weight; Actrapid, Novo Nordisk A/S, Bagsværd, Denmark) was infused over 5 min (from minute 20 to 25), while blood samples to measure glucose and insulin concentrations were collected for 3 h after glucose injection (19, 20).

### Sample Analyses

#### IVGTT

Plasma glucose concentrations were determined on an automated glucose analyser (YSI 2300 Stat Plus; YSI Life Sciences, Yellow Spring, OH, USA), and plasma insulin concentrations were determined by using electrochemiluminescence technology (Roche/Hitachi Cobas e411 immunochemistry analyzer; Roche Diagnostics, Indianapolis, IN). Whole-body insulin sensitivity (Si), the acute insulin response to glucose (AIRg), and the disposition index—which provides an assessment of the appropriateness of insulin secretion in relationship to peripheral insulin sensitivity (i.e., beta-cell function)—were determined by minimal modeling analysis of the glucose and insulin concentration data during the 3 h of the IVGTT with the MinMod Millennium software (26, 27). The minimal model is the simplest model that can account for the observed relationship between the glucose-insulin data after an IVGTT, and embodies two key concepts of glucoregulation: (i) once glucose is elevated by injection, it returns to baseline levels not only due to the action of insulin on peripheral glucose uptake, but also the effect of glucose itself to normalize its own concentration; and (ii) the effect of insulin on glucose disappearance exhibits a time delay. These concepts are described in two equations; one that relates glucose disappearance to glucose and insulin levels, and a second that describes the kinetics of insulin movement (28). We also calculated the HOMA-IR score as an index of whole-body insulin resistance, which is thought to reflect hepatic insulin action to a greater extent than muscle insulin action (29) (as opposed to the IVGTT-derived Si that predominantly reflects muscle insulin action).

#### Lipoprotein Profiling

Plasma concentrations of very low-density lipoprotein (VLDL), low-density lipoprotein (LDL), and HDL particles and subclasses were determined by using ^1^H-NMR spectroscopy on a Vantera Clinical Analyzer at LipoScience (LabCorp, Morrisville, NC) (30, 31). Lipoprotein particle concentrations and sizes were calculated as previously described using the LP4 algorithm (32). Concentrations of the following lipoprotein subclass categories were measured: very large VLDL (including residual chylomicrons, 90–240 nm), large VLDL (50–89 nm), medium VLDL (37–49 nm), small VLDL (30–36 nm), and very small VLDL (24–29 nm); large LDL (21.5–23 nm), medium LDL (20.5–21.4 nm), and small LDL (19–20.4 nm); and large HDL (9.6–13 nm), medium HDL (8.1–9.5 nm), and small HDL (7.4–8.0 nm). Average VLDL, LDL, and HDL particle sizes (diameter in nm) were computed as the sum of the diameter of each subclass multiplied by its relative mass percentage (32). NMR determinations of lipoprotein particle sizes correlate well with size estimations derived from conventional methods, e.g., gradient gel electrophoresis (32). Reproducibility of NMR determinations, expressed as the coefficient of variation (CV) of repeated measurements on the same samples, was 11% for total VLDL, 4% for total LDL, and 2% for total HDL particle concentrations, 4% for VLDL size, and 1% for LDL size and HDL size. Plasma triglyceride, cholesterol, and apolipoprotein B and A1 concentrations were determined by NMR; these values are highly correlated (*r* > 0.9) with the respective measurements from conventional lipid analysis (32).

### Statistical Analysis

A Kolmogorov–Smirnov test was performed to assess the normality of data distributions. For normally distributed data, differences between the two groups were evaluated by using the Student's unpaired *t*-test, and results are shown as means and SDs. For non-normal distributions, the Mann–Whitney *U*-test was used instead, and results are shown as medians and quartiles. Statistical significance was accepted at *p* ≤ 0.05. Statistical analysis was performed with SPSS version 23 (IBM SPSS, Chicago).

## Results

By design, the Si (insulin sensitivity) was ~50% lower in insulin-resistant than in insulin-sensitive subjects, whereas age, sex distribution and BMI were similar (Table 1). Insulin-resistant individuals had more body fat than insulin-sensitive ones. AIRg (insulin secretion) and fasting plasma insulin concentration were both ~50% greater in the insulin-resistant than the insulin-sensitive group, but the disposition index and fasting plasma glucose concentration were not different (Table 1), suggesting that greater insulin secretion in insulin-resistant subjects was able to compensate for much of the effect of insulin resistance on glucose homeostasis. Both glucose (4% greater) and insulin (53% greater) areas under the curve were significantly greater in insulin-resistant than in insulin-sensitive subjects (glucose: 19,916 ± 1,076 vs. 19,065 ± 1,099 mg·min/dl, respectively, *p* = 0.041; insulin: 7,200 ± 1,320 vs. 4,719 ± 1,217 mU·min/L, respectively, *p* = 0.00001). The HOMA-IR score was ~60% greater in the insulin-resistant than the insulin-sensitive group (Table 1).

By contrast, there were no significant differences between groups in resting blood pressure and heart rate (*p* ≥ 0.25), blood lipid profile (*p* ≥ 0.44) or lipoprotein subclass concentrations (*p* ≥ 0.30) and particle sizes (*p* ≥ 0.43; Table 2). All cardiovascular risk markers evaluated were well within normal range in both insulin-sensitive and insulin-resistant subjects (Table 2).

**Table 2:** Cardiovascular risk factor profile and lipoprotein subclass distribution and particle size in insulin-resistant and insulin-sensitive Asians.

	**Insulin- sensitive**	**Insulin- resistant**	***p-*value**
Systolic blood pressure (mmHg)	111 ± 7	111 ± 12	0.87
Diastolic blood pressure (mmHg)	70 ± 9	72 ± 6	0.53
Resting heart rate (bpm)	73 ± 12	77 ± 7	0.26
**Lipid profile**			
Triglyceride (mmol/L)	0.89 ± 0.42	0.87 ± 0.46	0.90
VLDL triglyceride (mmol/L)	0.73 ± 0.40	0.71 ± 0.44	0.90
Total cholesterol (mmol/L)	4.08 ± 0.84	3.92 ± 0.74	0.59
VLDL cholesterol (mmol/L)	0.51 ± 0.22	0.45 ± 0.20	0.44
LDL cholesterol (mmol/L)	2.17 ± 0.64	2.08 ± 0.57	0.69
HDL cholesterol (mmol/L)	1.39 ± 0.20	1.39 ± 0.34	0.97
Apolipoprotein B (nmol/L)	1281 ± 376	1234 ± 382	0.73
Apolipoprotein A1 (μmol/L)	46.7 ± 5.5	46.9 ± 9.4	0.95
**Lipoprotein particle concentration**			
VLDL particle concentration (nmol/L)	105.1 ± 46.2	90.5 ± 43.8	0.38
Very large VLDL	0.03 ± 0.05	0.03 ± 0.06	1.00
Large VLDL	1.81 ± 2.26	2.21 ± 2.67	0.66
Medium VLDL	13.4 ± 9.3	12.5 ± 8.0	0.78
Small VLDL	49.6 ± 22.2	41.7 ± 26.4	0.38
Very small VLDL	40.2 ± 32.4	34.1 ± 42.1	0.66
LDL particle concentration (nmol/L)	1087 ± 289	1091 ± 375	0.98
Large LDL	394 ± 200	326 ± 147	0.30
Medium LDL	269 ± 231	218 ± 151	0.48
Small LDL	424 ± 254	546 ± 430	0.35
HDL particle concentration (μmol/L)	18.3 ± 2.5	18.7 ± 2.9	0.68
Large HDL	3.3 ± 1.2	3.4 ± 1.8	0.92
Medium HDL	4.8 ± 0.9	4.5 ± 1.2	0.39
Small HDL	10.1 ± 2.9	10.8 ± 2.7	0.52
**Lipoprotein particle size**			
VLDL diameter (nm)	42.0 (39.6–45.6)	43.2 (39.5–48.8)	0.62
LDL diameter (nm)	21.2 (20.9–21.5)	21.1 (21.0–21.4)	0.43
HDL diameter (nm)	9.5 (9.3–9.7)	9.4 (9.0–9.7)	0.44

*Data are shown as mean ± SD or median (quartiles) for n = 15 each, and differences between groups were evaluated by Student's unpaired t-test or Mann–Whitney U-test, respectively*.

*VLDL, very low-density lipoprotein; LDL, low-density lipoprotein; HDL, high-density lipoprotein*.

## Discussion

We evaluated the lipoprotein subclass distribution and size in two groups of lean Asians with very different insulin sensitivity and found no significant differences in the plasma lipid profile, lipoprotein particle distribution, subclass concentrations, and particle sizes. Our results contradict the recurrent observation in individuals with overweight and obesity that abnormalities in lipid and lipoprotein metabolism coincide with insulin resistance (10–15).

In individuals with overweight and obesity, we and others have previously demonstrated that insulin resistance is associated with more large VLDL particles, leading to increased plasma triglyceride concentrations; and a shift of LDL and HDL subclass distributions from larger to smaller particles and therefore smaller average sizes (11–13). Furthermore, insulin sensitivity evaluated by using the hyperinsulinemic euglycemic clamp (11) and the IVGTT with minimal modeling (12) correlate directly with many abnormalities in lipoprotein subclass distributions, particle concentrations and average sizes. Conversely, among lean men and women (BMI <25 kg/m^2^), no significant differences in the traditional plasma lipid profile (fasting triglyceride and LDL and HDL cholesterol concentrations) were reported between insulin-resistant and insulin-sensitive groups, despite differences in insulin sensitivity (HOMA-IR) in the order of 50–140% (16–18). Our results extend these observations by demonstrating no differences in lipoprotein particle concentrations, subclass distributions, and particle sizes between insulin-resistance and insulin-sensitive lean subjects.

Only one study in non-obese subjects (BMI < 30 kg/m^2^) assessed lipoprotein subclass distribution and size by using ^1^H-NMR spectroscopy, in addition to the traditional lipid profile, and reported that the insulin-resistant group had more large VLDL, small LDL, and small HDL particles compared with the insulin-sensitive group, together with greater triglyceride and lower HDL cholesterol concentrations (33). However, both groups of non-obese subjects in that study had average BMI values in the overweight range (25–30 kg/m^2^) and moreover, the insulin-resistant individuals had significantly greater BMI than the insulin-sensitive ones, by ~2 kg/m^2^ (33). Similar differences in the plasma lipid profile (greater triglyceride and lower HDL cholesterol concentrations in non-obese insulin-resistant than insulin-sensitive subjects) were reported in a biracial cohort (African American and European American) (34), but here again, the BMI of the insulin-resistant group was significantly greater by ~2.2 kg/m^2^ than the BMI of the insulin-sensitive group, accompanied by significantly greater waist circumference as well (34). This makes interpretation of the reported differences in plasma lipid profile and lipoprotein subclass distributions difficult, as unfavorable changes in lipid and lipoprotein metabolism may have been due to greater whole-body (or central) adiposity rather than insulin resistance *per se*. We have argued recently that an important caveat of many studies comparing metabolic function and cardiometabolic risk factor profile between insulin-sensitive (or “metabolically healthy”) and insulin-resistant (or “metabolically unhealthy”) lean subjects is that the two groups often differ significantly in BMI, even if both fall within the normal-weight range (35). Such differences likely introduce significant bias because BMI is linearly associated with multiple adverse changes in cardiometabolic risk even within the normal body weight range (35). Therefore, the observed differences in lipid and lipoprotein metabolism between insulin-sensitive and insulin-resistant non-obese subjects in these studies (33, 34) may well be due to the different BMI between groups.

In our study, we carefully matched groups on age, sex, and BMI, and found no evidence of differences in lipid profile and lipoprotein subclass distribution between insulin-resistant and insulin-sensitive subjects. Our findings seem to contradict those from a recent study in lean Japanese subjects, matched for BMI, in whom insulin resistance at the level of adipose tissue (defined as low suppression of plasma fatty acid concentrations—and by extrapolation adipose tissue lipolysis—during exogenous insulin infusion) was accompanied by increased triglyceride and decreased HDL cholesterol concentrations (36). We speculate the different definition of insulin resistance (clamp-derived suppression of circulating fatty acids vs. Si from IVGTT in our study) is likely responsible for this discrepant observation on plasma lipid profile (lipoprotein particle concentrations and sizes were not evaluated in that study). Using adipose tissue lipolysis and plasma fatty acid availability to define insulin sensitivity and resistance (36) already sets the insulin-resistant group to have abnormalities in lipid metabolism (by definition), so differences in plasma lipid profile should not be unexpected. In our study, we used indices of insulin action on glucose metabolism (Si, but also HOMA) and found that insulin resistance is not accompanied by abnormalities in lipid and lipoprotein metabolism in the absence of obesity.

Our study has several strengths including the careful matching of the insulin-resistant and insulin-sensitive groups on age, sex, and BMI; the use of well-established methodologies to assess insulin sensitivity (IVGTT with minimal modeling) and lipoprotein subclass distribution (^1^H-NMR); and the standardization of pre-study diet and physical activity, which was achieved by having subjects spend the day prior to the metabolic tests in the laboratory while providing them with all foods consumed and controlling their physical activity. We have demonstrated previously that variation in dietary energy intake and exercise on the day before metabolic testing can affect both insulin sensitivity and plasma lipid and lipoprotein metabolism for at least a few days later (19, 37, 38). On the other hand, our sample size was relatively small and included only Asian participants of Chinese descent, hence we cannot rule out the possibility that we did not have adequate statistical power to detect small differences in lipoprotein profile, or that results may vary among other ethnicities. For instance, overweight and obesity in Asians may occur at lower BMI values than in Caucasians (around 23 and 27.5 kg/m^2^, respectively) (39). Two subjects in each group had a BMI > 23 kg/m^2^ but the groups were matched for BMI and, on average, mean BMI remained <22 kg/m^2^. Thus, we feel this does not confound our analysis nor does it affect our conclusions. Also, it is possible the difference in insulin action between our two groups was not large enough to have affected lipoprotein metabolism. However, our two study groups had very similar HOMA-IR values to those reported previously in insulin-sensitive and insulin-resistant individuals (0.9–1.6 and 1.7–2.5, respectively) (16–18), and also comparable IVGTT-derived Si values to those in overweight/obese subjects with normal glucose tolerance and prediabetes (3.8 and 1.6, respectively) (40). Therefore, the difference in insulin sensitivity between our lean subject groups (be it at the level of muscle or liver) was clinically relevant but not associated with abnormalities in lipoprotein metabolism. We also did not assess central adiposity in our subjects (e.g., visceral fat) or ectopic fat deposition (e.g., liver fat), which likely have a key role in mediating metabolic abnormalities among normal weight individuals (35). Finally, it is also possible that, because our subjects were relatively young on average, insulin resistance had not been instituted for a long enough time to cause changes in lipoprotein metabolism and subclass profile and distribution. We did not monitor our subjects longitudinally so we cannot ascertain the temporal nature of these relationships.

We conclude that in lean Chinese adults with normal glucose tolerance, insulin resistance is not accompanied by unfavorable changes in the plasma lipid profile and lipoprotein particle concentrations and sizes. This indicates a dissociation—which can be mechanistic or just temporal—between abnormalities in insulin action and in lipid and lipoprotein metabolism in the absence of obesity.

## Data Availability Statement

The raw data supporting the conclusions of this article will be made available by the authors, without undue reservation.

## Ethics Statement

The studies involving human participants were reviewed and approved by the Domain Specific Review Board of the National Healthcare Group in Singapore (protocol # 2016/00660). The patients/participants provided their written informed consent to participate in this study.

## Author Contributions

FM designed the study and secured research funding. CD, YC, ZC, JC, and ML conducted the experiments and contributed to acquisition of data. KT and FM performed statistical analyses and contributed to data interpretation. KT drafted the manuscript. All authors critically revised the manuscript and approval the final version submitted for publication.

## Conflict of Interest

The authors declare that the research was conducted in the absence of any commercial or financial relationships that could be construed as a potential conflict of interest. The reviewer SB declared a shared affiliation with one of the authors ML, to the handling editor at time of review.
